# Sinking skin flap syndrome with delayed dysautonomic syndrome—An atypical presentation^☆^

**DOI:** 10.1016/j.ijscr.2013.08.013

**Published:** 2013-09-08

**Authors:** Flávio Ramalho Romero, Marco Antônio Zanini, Luis Gustavo Ducati, Roberto Colichio Gabarra

**Affiliations:** a Division of Neurosurgery, Botucatu Medical School, São Paulo State University, UNESP, Botucatu, Brazil; b Hospital das Clínicas, UNESP, Botucatu, Brazil

**Keywords:** Sinking skin flap syndrome, Dysautonomic syndrome, Trefinated

## Abstract

**INTRODUCTION:**

Sinking skin flap syndrome or “syndrome of the trephined” is a rare complication after a large craniectomy, with a sunken skin above the bone defect with neurological symptoms such as severe headache, mental changes, focal deficits, or seizures.

**PRESENTATION OF CASE:**

We report a case of 21 years old man with trefinated syndrome showing delayed dysautonomic changes.

**DISCUSSION:**

Our patient had a large bone flap defect and a VP shunt that constitute risk factors to develop this syndrome. Also, there is reabsorption of bone tissue while it is placed in subcutaneous tissue. The principal symptoms of sinking skin flap syndrome are severe headache, mental changes, focal deficits, or seizures. Our patient presented with a delayed dysautonomic syndrome, with signs and symptoms very characteristics. Only few cases of this syndrome were related in literature and none were presented with dysautonomic syndrome.

**CONCLUSION:**

We reported here a very uncommon case of sinking skill flap syndrome that causes a severe dysautonomic syndrome and worsening the patient condition.

## 1 Introduction

Sinking skin flap syndrome or “syndrome of the trephined” is a rare complication after a large craniectomy, with a sunken skin above the bone defect with neurological symptoms such as severe headache, mental changes, focal deficits, or seizures.^1–5^ This phenomenon may result from atmospheric pressure gradient that may be aggravated by CSF diversion, CSF hypovolemia, dehydration, and position change.^1,2,6,7,11^

The syndrome of the sinking skin flap was introduced to explain neurological deterioration after decompressive craniectomy that is widely used in neurosurgical field for the relief of intractable intracranial hypertension in patients with head injury, acute stroke, and severe brain edema after intracranial procedure.^8–12^ The sinking skin flap syndrome may progress to “paradoxical herniation” as a consequence of the atmospheric pressure exceeding intracranial pressure and may eventually lead to neurological deterioration, coma or death.^1–4,12,13,9,14^ Therefore, it is important to understand pathophysiologic mechanism and treatment of this condition.

Dysautonomia is a distinct clinical syndrome, associated with a poorer functional outcome. Features of the syndrome include severe, paroxysmal increases in heart rate, respiratory rate, temperature, and blood pressure, with decerebrate or decorticate posturing, increased muscle tone, and profuse sweating. Although it is well recognized in the acute recovery phase after traumatic brain injury, a syndrome of prolonged episodes of autonomic abnormalities has been reported to follow a wide variety of cerebral pathologies. Delayed dysautomonic syndrome is a very uncommon presentation.^8–10^

We present a patient with dysautomonic syndrome and neurological deterioration with severe depression of the skull bone defect area resulting from decompressive craniectomy against traumatic brain swelling, developed after eighteen months of traumatic event.

## 2 Case report

**Fig. 1:**
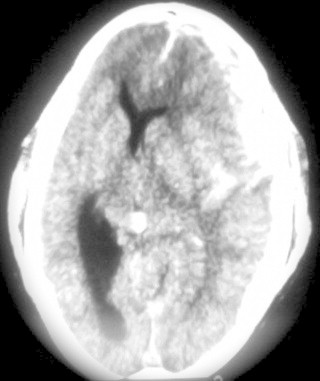
CT scan on admission showing extensive acute left subdural hematoma.

A 21-year-old man was transferred to our neurosurgical department in comatose state after car accident. Admission Glasgow Coma Scale score was 6, with left anisocoria. The brain computed tomographic (CT) scans showed extensive left acute subdural hematoma on the frontotemporoparietal region with significant mass effect and extensive midline shift to the right (Fig. 1). An emergency left frontotemporoparietal decompressive craniectomy for hematoma evacuation was performed with duraplasty to treat the important brain edema. The bone flap was placed in subcutaneous abdominal tissue. Postoperative follow-up brain CT scans showed the resolution of previous subdural hematoma and restoration of significant midline shift. He was transferred to intensive care unit and after some days developed infectious complications.

After a month, he developed hydrocephalus and a ventriculoperitoneal (VP) shunt was performed with a medium pressure valve. The patient received intensive care treatment with gradually recovered and was nearly alert enough to obey command. After three months the cranioplasty was performed. Previous cranial incision was opened, skin flap was rotated showing dura mater and duraplasty region and the bone flap was replaced and tightly fixed with nylon suture. After, skin incision was closed carefully. After more two days, he was discharged to rehabilitation program. The surgical scar was in good aspect, without any signs of possible complication.

**Fig. 2:**
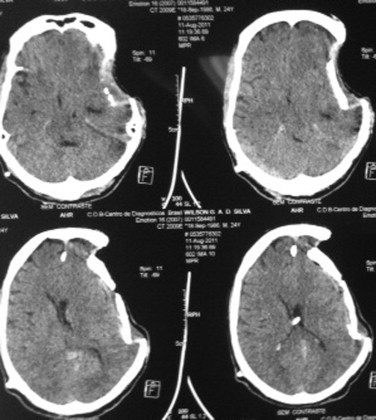
Patients CT scan exam after worsening in neurological condition.

**Fig. 3:**
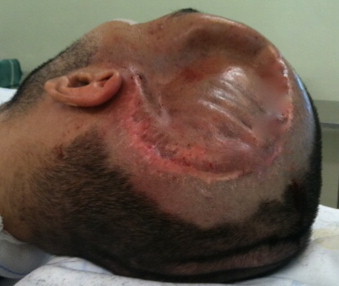
Patient after worsening in his neurological condition, before revision of VP system.

After fifteen months, he was in a very good state, walking with support and talking some sentences. The family noted that the bone flap was little depressed, but we opted to observe the evolution. Two months latter, he developed somnolence and paroxysmal increases in heart rate, respiratory rate, temperature, and blood pressure, with increased muscle tone and profuse sweating. Clinical exam and CT scan showed that the bone flap was very depressed through the brain tissue (Figs. 2 and 3).

We revised the VP shunt system and changed the medium pressure valve by higher pressures system. Also, clinical treatment was started to dysautonomic syndrome with bromocriptine, morphine sulfate and propranol. First, revision of cranioplasty was considered, but the bone flap was recovering the normal position day after day. So, cranioplasty aspect was observed and not revised. Patient recovered his previous neurological condition gradually in three weeks and the medications were stopped one by one. At this moment, the cranioplasty was in a good position. He was discharged in a good clinical and neurological condition to recovery the rehabilitation program. Six months latter, he improved his neurological condition, walking with minimal support and talking some sentences.

## 3 Discussion

Yamamura first reported the “trephined” or the “sinking skin flap” (SSF) syndrome in literature in 1977.^6^ It is a rare complication after a large skull bone defect that consists of a sunken skin above the bone defect with neurological symptoms such as severe headaches, mental changes, focal deficits, or seizures.^1,10–13,9^ The SSF may progress to “paradoxical herniation” as a consequence of the atmospheric pressure exceeding intracranial pressure and may eventually lead to coma and death.^1,2,4^

After that, many investigators tried to explain the pathophysiology of this condition. Some authors defended that atmospheric pressure to be directly transmitted to the intracranial cavity, causing inward shifting of the scalp over craniectomy site.^1–3,11–13,9,14^ Recently, authors proposed that a negative gradient between atmospheric and intracranial pressure, which is aggravated by changes in the CSF compartment following CSF hypovolemia to be the mechanism of neurological deterioration after craniectomy.^1–3,12,9^ The CSF drainage in a patient suffering from hydrocephalus, or meningitis exacerbates this effect by creating a pressure gradient through craniectomy site. Also, prolonged dehydration and up-right position may precipitate this phenomenon.^1–3^ Our patient had a large bone flap defect and a VP shunt that constitute risk factors to develop this syndrome. Also, there is reabsorption of bone tissue while it is placed in subcutaneous tissue. Some author described almost 20% of bone lost after three months.^2,6,8^ This fact could difficult the bone flap fixation in cranioplasty margin, creating great mobility in the bone defect area, increasing the risk of sinking skin flap syndrome.

The post-traumatic hydrocephalus is finding in patients that undergoing decompressive craniectomy after head injury in 10.1–28.6%.^2–4,9^ Although the obvious causal relationship between decompressive craniectomy and hydrocephalus remains unclear, the disturbance of CSF flow around the cerebral convexities may be the possible mechanism. In the context of hydrocephalus after decompressive craniectomy, it is difficult for neurosurgeon to decide whether CSF diversion can be safely performed.^12,13,9,14^

The principal symptoms of sinking skin flap syndrome are severe headache mental changes, focal deficits, or seizures.^1,10–13,9,14^ Our patient presented with a delayed dysautonomic syndrome, with signs and symptoms very characteristics. Only few cases of this syndrome were related in literature^1,4,5,7,11,14^ and none were presented with dysautonomic syndrome.

The goal of treatment in sinking skin flap syndrome is restoration of the pressure exerted by depression of craniectomy site.^1–3,10–13,9,14^ Several authors suggested that severe CSF hypovolemia after craniotomy may produce a dramatic herniation syndrome that is completely reversed by the Trendelenberg position.^9,14^ Also, it was reported that intrathecal saline infusion reverses impending transtentorial herniation in patients with decline of mental status due to intracranial hypotension.^12^ However, conservative management of the syndrome of the sinking skin flap with neurological deterioration is largely ineffective.

Cranioplasty is the principal surgical treatment that could improves the neurological deficits by a decrease in local intracranial pressure, and correction of abnormal CSF dynamics.^11–13,9,14^ Other theory suggested that cranioplasty might affect postural blood flow regulation, cerebrovascular reserve capacity and cerebral glucose metabolism.^9^ However, the cranioplasty for severe sinking at the skull defect may result in the dysfunction of the underlying brain, risk of fluid collection and hematoma formation in the subdural space due to the large dead space.^1–3,14^ Our patient performed the cranioplasty before the development of sinking skin flap syndrome and the bone flap was depressed together the skin flap. So, we considered that cranioplasty was not the main cause. Otherwise, our concerning was the treatment of the over-draining syndrome caused by VP shunt.

The safe and effective surgical methods to expand the scalp depression and to eliminate the dead space in the context of VP shunt are temporary occlusion or removal of shunt device before cranioplasty.^1–3^ We choose change the VP system by a higher pressures valve to improve the scalp depression and then correct spontaneously the bone flap.

## 4 Conclusion

We reported here a very uncommon case of sinking skill flap syndrome that causes a severe dysautonomic syndrome and worsening the patient condition. Our option was to treat the patient with VP shunt revision and change the system by a higher pressures valve, with good resolution.

## Conflict of interest

None.

## Funding

None.

## Ethical approval

Written informed consent was obtained from the patient for publication of this case report and accompanying images. A copy of the written consent is available for review by the Editor-in-Chief of this journal on request.

## Author contributions

Flávio R. Romero contributed to the study design, data collections, data analysis, writing and review. Marco A. Zanini and Roberto C. Gabarra contributed to the writing and review. Luis G. Ducati contributed to the data collections and review.
